# The Role of Artificial Intelligence in Anterior Cruciate Ligament Injuries: Current Concepts and Future Perspectives

**DOI:** 10.3390/healthcare12030300

**Published:** 2024-01-24

**Authors:** Luca Andriollo, Aurelio Picchi, Rudy Sangaletti, Loris Perticarini, Stefano Marco Paolo Rossi, Giandomenico Logroscino, Francesco Benazzo

**Affiliations:** 1 Robotic Prosthetic Surgery Unit—Sports Traumatology Unit, Fondazione Poliambulanza Istituto Ospedaliero, 25124 Brescia, Italy; rudy.sangaletti@poliambulanza.it (R.S.); loris.perticarini@poliambulanza.it (L.P.); stefano.rossi@poliambulanza.it (S.M.P.R.); francesco.benazzo@poliambulanza.it (F.B.); 2 Department of Orthopedics, Catholic University of the Sacred Heart, 00168 Rome, Italy; 3 Unit of Orthopedics, Department of Life, Health and Environmental Sciences, University of L’Aquila, 67100 L’Aquila, Italy; aurelio.picchi@graduate.univaq.it (A.P.); giandomenico.logroscino@univaq.it (G.L.); 4 Biomedical Sciences Area, IUSS University School for Advanced Studies, 27100 Pavia, Italy

**Keywords:** artificial intelligence, machine learning, navigation, anterior cruciate ligament, deep learning, knee injuries

## Abstract

The remarkable progress in data aggregation and deep learning algorithms has positioned artificial intelligence (AI) and machine learning (ML) to revolutionize the field of medicine. AI is becoming more and more prevalent in the healthcare sector, and its impact on orthopedic surgery is already evident in several fields. This review aims to examine the literature that explores the comprehensive clinical relevance of AI-based tools utilized before, during, and after anterior cruciate ligament (ACL) reconstruction. The review focuses on current clinical applications and future prospects in preoperative management, encompassing risk prediction and diagnostics; intraoperative tools, specifically navigation, identifying complex anatomic landmarks during surgery; and postoperative applications in terms of postoperative care and rehabilitation. Additionally, AI tools in educational and training settings are presented. Orthopedic surgeons are showing a growing interest in AI, as evidenced by the applications discussed in this review, particularly those related to ACL injury. The exponential increase in studies on AI tools applicable to the management of ACL tears promises a significant future impact in its clinical application, with growing attention from orthopedic surgeons.

## 1. Introduction

Technical developments in orthopedic surgery have resulted in two main contributions: the integration of artificial intelligence (AI) into decision support systems for diagnosing and treating orthopedic conditions, and the adoption of robotic surgery in surgical procedures.

The inception of AI dates back to 1956 when Professor John McCarthy first introduced the concept. Since then, the notion of AI has undergone a transformative evolution, revealing deep learning (DL) and evolutionary networks that can replicate the functions of human neuron cells [1].

The remarkable progress in data aggregation and DL algorithms has positioned AI and machine learning (ML) to revolutionize the field of medicine [2,3]. AI represents the fourth industrial revolution and is the upcoming frontier in medicine, with the potential to revolutionize the field of orthopedics and sports medicine. However, the comprehensive understanding of fundamental principles and the integration of applications are still in their early stages [4,5].

AI is becoming more and more prevalent in the healthcare sector, and its impact on orthopedic surgery is already evident in several fields. AI has found applications in many aspects of orthopedic surgical care, including its integration into the diagnosis and classification of fractures, assessment of risks and prediction outcomes, intraoperative navigation, and robot-assisted surgery.

Recent research efforts aimed at integrating AI into orthopedic surgery and sports medicine have shown significant potential. These applications hold promise in predicting the risk of athlete injuries, interpreting advanced imaging, assessing patient-reported outcomes, reporting value-based metrics, and enhancing the overall patient experience [6,7]. Emerging technologies like AI demand ownership, leverage, and application by orthopedic surgeons in order to improve the care provided to patients [8]. There are still issues to be addressed with regard to regulation, ethical application, and AI’s clinical advantage over conventional statistics and decision-making [9,10].

The knee is involved in nearly half of all injuries in orthopedic sports pathology. Anterior cruciate ligament (ACL) tears are the most common injury among these, accounting for up to 78% of all sports-related knee pathology [11,12].

Despite the ongoing debate in the literature regarding ACL reconstruction, encompassing various surgical techniques, graft and fixation devices, and rehabilitation approaches, the procedure is considered safe. It demonstrates reproducibility among knee surgeons, patient satisfaction, and positive outcomes, even in individuals with high functional demands [13,14]. Consequently, the application of AI, which has shown a significant impact in other orthopedic domains for patient care, has not fully achieved its potential in this context. Moreover, current barriers to the adoption of AI and ML in the treatment of knee injuries include regulatory hurdles, data privacy concerns, and the imperative need for thorough validation of AI models within the orthopedic field.

In the management of ACL tears, AI and ML can play a role in improving diagnostics, accurately predicting individuals at risk of ACL injury or re-injury, identifying complex anatomic landmarks during surgery, and optimizing pain control and postoperative rehabilitation protocols [15,16]. However, interest and understanding of AI and ML in knee injuries are still little-known and underutilized. 

This review aims to examine the literature that explores the comprehensive clinical relevance of AI-based tools utilized before, during, and after ACL reconstruction, specifically focusing on the use of AI, ML, and DL, also describing their conceptual differences. The goal is to address and enhance the detection, treatment, and rehabilitation of individuals with ACL injuries.

## 2. AI Tools

AI empowers machines to execute tasks through algorithms driven by pattern recognition and self-correction, utilizing extensive data to refine options and prevent errors. The four essential elements for implementing AI in medicine include big datasets, high-performance computers, cloud computing infrastructure, and the development of open-source algorithms. The utilization of AI in healthcare is continually growing, and its influence on orthopedic surgery is already evident in various domains. These include recognizing medical images, predicting risks, and aiding in clinical decision-making processes [7,8,16].

ML emerged soon after the advent of AI in 1959 as a method to achieve AI objectives. ML algorithms are capable of learning from data by modifying their internal parameters and strengthening relevant connections, thereby improving the accuracy of a specific model. This learning process in ML entails an incremental optimization of a mathematical model [8,17].

DL represents a more advanced form of ML. It is capable of conducting unsupervised learning using unstructured and unlabeled data, by filtering out data input from less relevant variables for targeted prediction. DL is inspired by the neuronal connections in the human brain and operates through algorithms called artificial neural networks [2,8].

**Figure 1:**
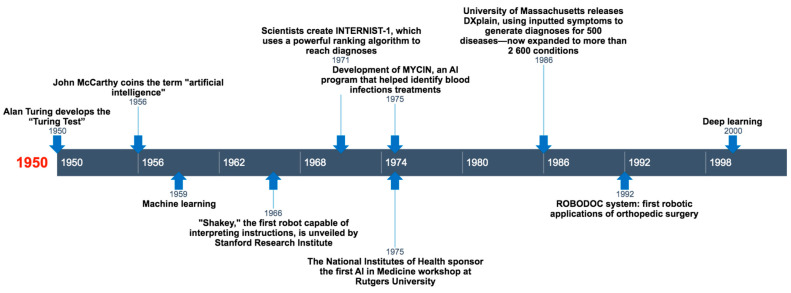
Timeline outlining the major historical steps that have led to the development of artificial intelligence (AI) up to the advancement of deep learning, with significant impact in the history of medicine and orthopedics.

In Figure 1, some significant steps in the impact of AI from its inception to DL are illustrated.

AI, ML, and DL offer a variety of application models in the orthopedic field, including the random forest model, support vector machine, multilayer perceptron, convolutional neural network (CNN), alternating decision trees, and recurrent neural network [4,15].

**Figure 2:**
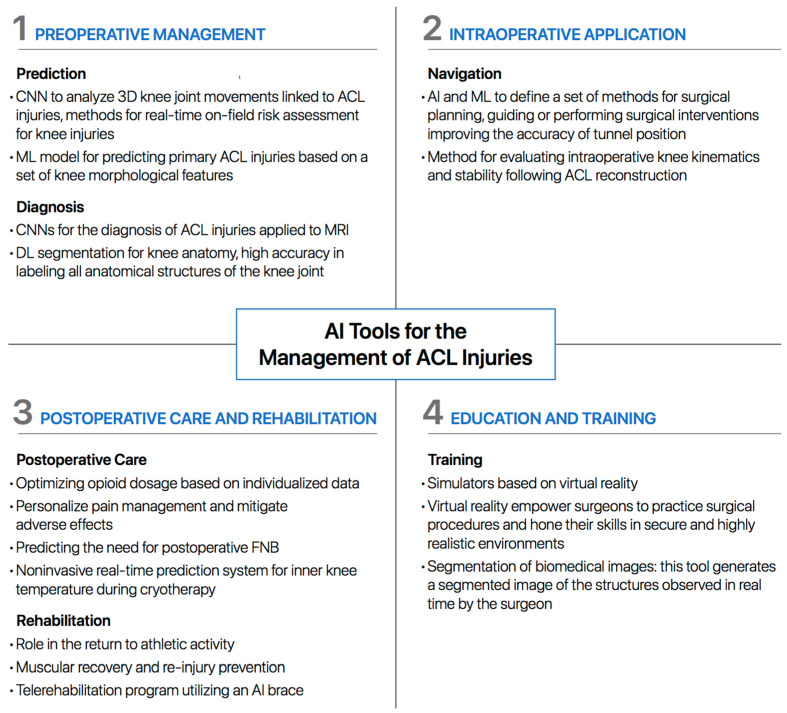
Key applications of AI tools in the management of ACL injuries, presented and discussed in the following sections (AI = artificial intelligence; CNN = convolutional neural network; ACL = anterior cruciate ligament; ML = machine learning; MRI = magnetic resonance imaging; DL = deep learning).

Figure 2 illustrates the main points of application of AI tools in the management of ACL injuries.

## 3. Preoperative Management

### 3.1. Prediction

AI tools and ML have enabled the development of algorithms to predict ACL injuries. Pedoia et al. developed a statistical shape modeling based on 3D magnetic resonance imaging (MRI). This model enables the extraction and comparison of the tibia and femur shapes in patients, both with and without acute ACL injuries [18]. It is known that certain features such as intercondylar width and posterior tibia slope are correlated with a higher risk of injury. However, this statistical shape modeling offers significant potential to measure these features, providing a detailed account of the surfaces that depict complex 3D deformations.

Johnson et al. have developed a DL-based system aimed at acting as an early alert mechanism for monitoring athlete workload and the risk of knee injuries by accurately capturing on-field 3D knee joint movements [19]. The algorithm is based on a pretrained CNN that analyzes 3D knee joint movements linked to ACL injuries. This is performed through marker-based motion capture as athletes engage in three sports-related activities: walking, running, and sidestepping. According to the authors, this DL-based system represents an initial stage toward establishing techniques for assessing the risk of knee injuries, including ACL tears, in real time during athletic events.

Taborri et al. developed an approach based on AI to quantify the risk of ACL injury, assessing leg stability, mobility, and load absorption capacity following a jump, utilizing inertial sensors and optoelectronic devices [20]. The athlete’s risk factor was determined using the Landing Error Score System, which showed a strong correlation for predicting ACL injury.

Tamini et al. employed supervised ML models to construct a predictive mathematical model for primary ACL injuries, using a set of knee morphological characteristics [21]. Preoperative MRI scans were utilized to measure the anteroposterior lengths of the medial and lateral tibial plateaus, as well as the lateral and medial bone slope, lateral and medial meniscal slope, and lateral and medial menisci. An AI-based prediction tool was developed with Matlab R2019b software, employing an algorithm to formulate the predictive model. The AI prediction model for primary ACL injury achieved a testing accuracy of over 90%.

### 3.2. Diagnosis

Injuries to the ACL, as well as other ligaments and menisci, are highly prevalent and are commonly diagnosed using knee MRI [22]. AI, particularly DL, has become a widely studied tool to enhance the capabilities of radiologists in various clinical applications [23,24]. Most DL algorithms designed to identify and describe internal derangement in MRI images have primarily focused on the knee joint. This emphasis can be attributed to the frequent occurrence of knee MRI exams, generally high image quality, significant clinical relevance, relatively straightforward anatomical structures, standardized positioning, and a well-defined set of common injuries [25]. 

The diagnosis is mainly based on expert clinical examinations to evaluate the stability of the knee. Both the Lachman test and the Pivot Shift test are clinical methods that show high sensitivity and specificity for identifying a complete tear of the ACL [26]. In addition to confirming an ACL tear, the main advantages of MRI include characterizing the tear type for surgical decision-making and diagnosing concomitant knee injuries.

In their pioneering study on machine learning models for diagnosing knee ligament injuries in 2017, Štajduhar et al. appraised two decision-support models that could distinguish between less severe ACL injuries not necessitating surgery and complete ACL tears requiring surgical intervention, based on sagittal plane MRI images of the human knee [27]. The process included extracting a histogram of oriented gradients (HOG) and gist descriptors from regions of interest around the cruciate ligament area. They tested two machine learning models, support vector machine (SVM) and random forests, in conjunction with both methods of feature extraction. To evaluate the model’s generalization, they applied stratified 10-fold cross-validation repeatedly and calculated the area under the curve (AUC) score. Experimental results indicate that a linear-kernel SVM trained on HOG descriptors exhibited superior generalization properties, achieving an AUC of 89% in diagnosing partial-thickness tears and 94% for complete tears. This semi-automated method underscored the potential of computer-assisted decision support in the semi-automated diagnosis of ACL injuries in a clinical setting.

Numerous completely automated investigations followed, demonstrating progressively positive outcomes from enhanced algorithms. In a study published in 2018 by Bien et al., the goal was to develop a deep CNN, MRNet, for interpreting knee MRI exams, with a focus on detecting a variety of knee injuries, including ACL tears [28]. The dataset included 1370 knee MRI exams and the network employed sagittal T2-weighted, coronal T1-weighted, and axial PD-weighted MRI images. The model achieved high accuracy with AUC values of 97% for diagnosing ACL tears. The study found that the model’s performance was comparable to unassisted general radiologists in detecting abnormalities, while clinical experts exhibited improved specificity in identifying ACL tears when assisted by the model. In fact, radiologists working with the support of the DL algorithm showed an increased sensitivity of 5%. The research suggests that DL models can enhance the performance of clinical experts in interpreting medical imaging, emphasizing the potential for improved diagnostic accuracy.

In a study published by Liu et al. in 2019, the feasibility of employing an automated DL-based approach for detecting ACL tears in knee MRI was assessed, with arthroscopy serving as the reference standard [29]. Employing sagittal fat-suppressed proton density and sagittal fat-suppressed T2-weighted images from a 3-Tesla (T) MRI, the DL approach demonstrated a sensitivity and specificity of 0.96 each, comparable to clinical radiologists (sensitivity: 0.96–0.98; specificity: 0.90–0.98). No significant difference in diagnostic performance was observed between the CNN and clinical radiologists, with an overall high diagnostic accuracy (AUC = 98%).

Other studies published in the same year assessed various CNNs for diagnosing full-thickness ACL tears. Richardson evaluated the efficacy of a CNN as a substitute for human readers in a protocol optimization study, using sagittal images in both fat-saturated (FS) and non-fat-saturated (NFS) conditions [30]. The results showed high performance with receiver operating characteristic AUC values of 99.8% for NFS and 99.9% for FS. While both FS and NFS demonstrated excellent sensitivity and specificity, FS sensitivity was statistically superior. Another study, conducted by Chang et al., assessed the feasibility and additional benefits of specific network architectures in employing DL for the MRI detection of complete ACL tears in sports injuries [31]. The evaluation was based on coronal proton density-weighted MRI images without fat suppression. Different CNN architectures were employed, with variations in input field-of-view and dimensionality. The model using a five-slice dynamic patch-based sampling algorithm achieved over 96% test set accuracy, showcasing the effectiveness of a customized 3D DL approach for detecting complete ACL tears in MRI scans.

Afterward, in 2020, studies on CNN applied to the diagnosis of ACL injuries have increased, confirming their promising results. In research conducted by Zhang et al., a 2D sagittal proton density-weighted spectral attenuated inversion recovery sequence at both 1.5 Tesla and 3.0 Tesla was employed [32]. A CNN based on the architecture of 3D DenseNet was constructed and tested alongside two other algorithms (VGG16 and ResNet). The customized 3D DL architecture achieved a sensitivity of 98%, a specificity of 94%, and an accuracy of 96%, outperforming ResNet (95%) and VGG16 (86%). Another study by Germann et al. conducted a study detailing a tailored approach for the automatic identification of ACL tears, which was tested using coronal and sagittal fat-suppressed fluid-sensitive MRI images, with arthroscopic surgery serving as the reference standard [33]. In tests on a uniform internal dataset that included MRI scans at both 1.5 Tesla and 3.0 Tesla, the CNN exhibited high accuracy, with a sensitivity of 99%, a specificity of 94%, and an AUC of 97%. However, when the CNN was evaluated on a more varied external dataset containing 234 knee MRI exams from over 50 different institutions, there was a drop in diagnostic accuracy, with the system achieving a sensitivity of 93%, a specificity of 87%, and an AUC of 90%.

Until that time, the majority of CNNs focused on a binary classification of ACL as intact or torn. However, in 2020, Namiri et al. introduced a multi-class CNN that adopted a more comprehensive method by categorizing the severity of ACL injuries into four different patterns: intact, partial-thickness tear, full-thickness tear, and post-reconstruction ACL graft [34]. This CNN showed high accuracy, with a sensitivity range of 97% to 100% and a specificity of 100% for identifying ACL grafts. For intact ACLs, the CNN achieved sensitivities between 89% and 93%, with specificities ranging from 88% to 90%. In the case of full-thickness ACL tears, the CNN showed sensitivities of 76% to 82% and specificities of 94% to 100%.

Since 2021, there has been an exponential increase in studies on custom architecture CNNs for the diagnosis of ACL injuries applied to MRI, and currently there are various DL models developed, such as VGG16, VGG19, U-Net, AdaBoost, XGBoost, Xception, MRPyrNet, Inception ResNet-v2, RadImageNet, and Inception-v3 DTL [35,36,37,38,39,40,41,42,43,44,45,46,47,48,49,50,51]. Awan et al. introduced a method that utilizes a tailored 14-layer ResNet-14 configuration of a CNN, which processes data in six distinct directions. This approach incorporates techniques of class balancing and data augmentation [35]. The AUCs for intact ACLs, partial tears, and complete ruptures were reported as 98%, 97%, and 99.9%, respectively. Li et al. conducted a study emphasizing the significant advantage of DL-based MRI sagittal plane detection in diagnosing ACL injury [35]. The method achieved high sensitivity (96.78%), specificity (90.62%), and accuracy (92.17%), with results comparable to those obtained through arthroscopy, indicating no substantial difference.

Recently, algorithms have been developed on increasingly larger populations. Minamoto et al. specifically discussed the development of an algorithm designed to detect ACL ruptures, utilizing a large dataset of nearly 20,000 MRI scans [44]. The algorithm demonstrated a notable performance with AUC values reaching 0.939, a sensitivity of 87%, and a specificity of 91%. Notably, this algorithm underwent testing on external populations from different countries, where it consistently exhibited strong performance with AUC values of 0.962 and 0.922, respectively.

The use of DL has been evaluated not only in diagnosing ACL injuries but also in recognizing the anatomical structures of the knee. In a study published in 2023 by Kulseng et al., they explored the application of DL segmentation for knee anatomy, specifically targeting 13 anatomical classes, utilizing an MRI protocol consisting of four 3D pulse sequences [52]. The DenseVNet neural network was employed, and five input combinations of sequences were trained. The DL network demonstrated high accuracy in labeling all anatomical structures of the knee joint (bone medulla, PCL, ACL, muscle, cartilage, bone cortex, arteries, collateral ligaments, tendons, meniscus, adipose tissue, veins, and nerves), showcasing potential clinical utility for preoperative evaluation and pathology detection.

## 4. Intraoperative Application

The intraoperative application of AI and ML has led to the development of navigation systems and anatomical segmentation. 

Navigation in ACL surgery refers to the use of computer technology for the development of procedures for surgical planning and guiding or performing surgical interventions. This technology enhances the precision of tunnel placement and facilitates the evaluation of kinematics and stability after ACL reconstruction (ACLR) [53,54].

In ACL reconstruction, navigation can be achieved through two approaches: image-based and image-free [55]. The image-based method employs either pre-operative computed tomography (CT) scans or intra-operative X-ray fluoroscopy, which provide real-time imaging during the surgical procedure but also expose the patient to ionizing radiation. On the other hand, the image-free technique usually relies on 3D bone morphing technology that utilizes an optical tracking system without the need for radiation exposure.

The literature cites a variety of systems, the most prevalent being PRAXIM-Medivision (La Tronche, France), Orthopilot (BrainLab, Munich, Germany), Vectorvision (BrainLab, Munich, Germany), and KneeNav (Pittsburgh, PA, USA), among others. Specifically for kinematic analysis, non-invasive inertial sensors that attach to the skin, suitable for clinical use like KiRA (Orthokey, Florence, Italy), have been developed [53].

The initial use of intraoperative navigation in arthroscopy was primarily directed toward optimizing tunnel positioning in ACLR to enhance graft kinematics and isometry. This is crucial as tunnel malposition significantly contributes to ACLR graft failure, emphasizing the value of navigation in improving the precision of tunnel placement [56].

Numerous studies have demonstrated that navigation can enhance the precision of anatomical tunnel orientation and placement in ACLR surgery when compared to conventional arthroscopic tunnel placement, including comprehensive systematic reviews [53,54,57,58,59]. The primary benefits are observed in the femoral tunnel and in surgeries performed by less experienced surgeons [60,61]. 

Another highly valuable application of the navigator during ACLR is when preserving remnants in ACL surgery, as these remnants can impact the visualization of the footprints [62]. Additionally, it proves beneficial in revision ACLR surgeries, where the presence of previous tunnels and fixation devices can make the accurate placement of the new graft more challenging [63,64].

The main advantages of using the navigation system include the accuracy and reproducibility of the femoral tunnel. These systems can be used to improve safety, minimize the risk of a short femoral tunnel, and prevent posterior wall breakage [55,65].

Navigation has emerged as the method of choice for intraoperative assessment of knee kinematics, as it enables a quantitative evaluation of the knee joint’s multidirectional laxity [15,54]. Its use has indeed been employed for evaluations of anteromedial vs. trans-tibial reconstruction, single bundle vs. double bundle, and reconstruction with the addition of an anterolateral tenodesis [66,67,68,69,70].

However, despite the usefulness of intraoperative navigation, both for the quality of bony tunnels and for kinematic assessments, its clinical use is currently very limited, with almost exclusive application in the realm of research [53]. Indeed, the surgical application of current systems can require increased exposure to imaging radiation and is more invasive, time-consuming, and expensive [63,71].

## 5. Postoperative Care and Rehabilitation

ML and AI are playing an increasingly significant role in postoperative care and rehabilitation after ACLR, offering the possibility to personalize and optimize treatments [15].

### 5.1. Postoperative Care

The potential risks brought about by opioid usage in post-operative pain have focused research attention on the management of opioids [72]. The exploration of factors influencing postoperative opioid administration, especially in the aftermath of trauma, addresses a prevalent challenge of opioid misuse [73]. This issue is marked by frequent complications, and it necessitates a proactive approach. In this context, AI and ML emerge as pivotal tools [74]. Utilizing these technologies enables the development of predictive models, optimizing opioid dosage based on individualized data. Healthcare professionals can identify at-risk patients, personalize pain management, and mitigate adverse effects by using AI and ML, thus revolutionizing postoperative care. This transformative potential underscores the significance of integrating AI and ML into the medical landscape to enhance patient outcomes and avoid the complications associated with opioid use in the context of postoperative pain [75]. Bharat et al. studied big data and ML to prevent and monitor opioid overdoses, addressing challenges and risks. The research group relates that collaboration between pharmaceutical agencies is essential to improve access to existing resources and implement predictive models that personalize the diagnosis, prognosis, and treatment recommendations [76]. ML predictive analysis can estimate the ideal dosage of drugs needed for pain control based on previous data. Anderson et al. utilized ML methods, including a gradient-boosting machine, to predict prolonged opioid use following ACLR. By analyzing Military Health System data on 10,919 patients, they developed and validated four models, with the gradient-boosting machine exhibiting the highest predictive power. Key features influencing opioid use included preoperative morphine equivalents, deployment time, age, and race. The model offers a clinical decision-support tool for identifying at-risk patients and preventing opioid overuse [77]. However, the lack of details and transparency in creating ML models limits the usefulness of the research. Improvements in documentation and sharing of source code are recommended to advance in this crucial healthcare field [78].

The immediate postoperative period in knee surgery includes the option to perform a femoral nerve block (FNB) for pain management [79]. The FNB, often combined with the sciatic nerve block, demonstrates the most opportunities for decreasing opioid consumption and alleviating pain severity [80]. Tighe et al. present an ML approach for predicting the need for postoperative FNB following ACLR. Analyzing perioperative data from 349 patients, ML classifiers, including logistic regression and tree algorithms, outperformed traditional methods, achieving a high area under the receiver operating curve. Despite overfitting concerns, ML shows promise in predicting severe postoperative pain and the necessity of peripherical nerve block, highlighting its potential over conventional statistical methodologies in medical data analysis [81]. 

Cryotherapy, in addition to being an effective method to contrast postoperative inflammation, pain, and swelling, represents a valuable resource in the field of pain management and rehabilitation. This technique, utilizing therapeutic cold, can help limit the inflammatory response, alleviate discomfort, and promote swift recovery after surgery [82]. Rashkovska et al. examined the regulation of temperature in cryotherapy [83]. The authors propose a noninvasive real-time prediction system for inner-knee temperature during cryotherapy after ACLR, utilizing computer simulations and ML. Model trees, with input from skin sensors, show excellent predictive accuracy and meet real-time response requirements, validated with in vivo patient temperature data collected during cryotherapy with specific sensors.

### 5.2. Rehabilitation

Post-operative rehabilitation plays a fundamental role in the return to athletic activity, particularly for athletes but also for all patient categories undergoing ACLR. It contributes to muscular recovery and re-injury prevention, while also supporting psychological recovery, ensuring that athletes regain confidence in their movements and achieve performance safely and effectively [82]. Some exercises performed during the return-to-play phase can be recorded and measured using specific parameters. Corban et al. studied the drop vertical jump (DVJ) using a motion capture system (Microsoft Kinect V2). The study revealed that an increase in peak coronal angles and a decrease in peak sagittal angles during the DVJ were associated with a higher risk of noncontact ACL injuries [84].

Dagget et al. retrospectively studied a motion capture system, analyzing algorithm-derived scores to objectively assess mobility, alignment, and readiness in post-ACLR patients. An innovative 3D motion capture system (DARI Motion, Overland Park, KS, USA), cleared by the Food and Drug Administration (FDA), was used to obtain biomechanical parameters of each body motion [85]. The findings suggest that assessing multiple biometric variables is crucial to avoid relying solely on super-compensatory mechanisms and underscores the potential of non-invasive motion capture technology in enhancing the objectivity and frequency of progress assessments for a more tailored and effective rehabilitation approach. The research highlighted challenges in monitoring progress due to the absence of established guidelines or objective assessment methodologies.

Strength deficits at specific angles and power, especially near full extension, indicate the need for comprehensive rehabilitation [86]. Richter et al. utilized a data-driven approach without expert knowledge, achieving over 70% accuracy in predicting movements post-ACLR, in contralateral limbs, and in healthy controls, but not all subjects exhibit a ‘true normal’ movement and classifiable pattern [87]. While many studies explore the association between movement patterns and athletic performance on injury risk, evidence-based guidelines are insufficient to define movement deficits or normal ranges of motion [88]. Accessible motion capture systems emerge as practical and cost-effective options for large-scale screening of ACL re-injury risk during the return-to-play phase, and this could be integrated with ML and AI systems. 

The persistence of the risk of re-injury after ACLR is a key concept, requiring careful assessment to ensure a safe and sustainable return to sports. The risk factors associated with revisions or graft re-rupture after reconstruction are numerous. The careful identification and management of these factors are crucial to reduce the risk of revisions or new injuries after ACLR [89]. An effective tool could be applied in this context to support clinical practice and decrease the re-injury rate. Pillitteri et al. investigated the correlation between internal load and external load, and their impact on injury risk prediction using ML approaches. The review shows an association between external load, internal load, and re-injury risk, highlighting the effectiveness of the ML approach [90]. Other predictive models about re-injury have been studied.

Martin et al. externally validated an ML model developed using data from the Norwegian Knee Ligament Register (NKLR) and Danish Knee Ligament Registry (DKLR) to predict the risk of ACL revision [91]. The model, applied to patients from the DKLR, demonstrated similar concordance but poorer calibration at one and five years post-primary surgery compared to the NKLR test data [92]. Despite dissimilarities in surgical techniques and injury characteristics between registries, the NKLR algorithm exhibited consistent performance. A calculator has been developed to estimate the risk of ACL revision, enabling risk stratification at the point of care based on only five variables [92]. This marks the first external validation of an ML model for predicting ACL revision, supporting its potential use in diverse patient populations. Martin et al. recently introduced an ML model that relates registries [93]. The combined ML examination of data from the NKLR and DKRR enabled the prediction of the risk of ACL reconstruction revision with moderate accuracy. Despite this, the algorithms generated were not as user-friendly and did not demonstrate better accuracy than the earlier model that was exclusively based on NKLR patient data, even though it analyzed a patient cohort of nearly 63,000. This suggests that merely increasing the number of patients in existing knee ligament registers may not enhance predictive capability. The observed ceiling effect could prompt future modifications, aiming to improve the inclusion of variables for more effective predictions. The research stimulates the need to create national registries and increase the number of patients. 

In response to the impossibility of in-person rehabilitation during the COVID-19 pandemic, a telerehabilitation program utilizing an AI brace was implemented for patients undergoing ACLR. The tele-AI patients, receiving remote rehabilitation, demonstrated superior short-term outcomes with higher International Knee Documentation Committee scores (IKDC) and Knee Injury and Osteoarthritis Outcome Scores (KOOS) at various intervals compared to the face-to-face patients undergoing hospital-based rehabilitation [94]. Notably, the tele-AI group exhibited a higher Tegner Activity Scale (TAS) after one year. This suggests that home-based telerehabilitation, especially during the pandemic, may offer effective and comparable clinical results to traditional in-person rehabilitation [95].

## 6. Education and Training

The growing importance of computer-assisted surgery has not only brought about fundamental changes in the skills required by surgeons but has also spurred the development of innovative educational methods [96]. Virtual reality (VR) simulators are designed to provide surgeons with a platform where they can refine their surgical techniques and improve their expertise in a safe, diverse, and highly realistic setting [97,98]. For these simulators to be fully effective as educational instruments, they need to have the capability to automatically assess performance and provide feedback. The latest advancements in this area depend on the application of DL [99]. 

Module-based simulation training offers additional training time and enhances technical skills. Simulation training enables individuals to refine their skills in a less stressful environment, all without posing any risk to patient safety [100]. The most widely used VR simulator in the field of knee arthroscopy is VirtaMed Arthro (Zurich, Switzerland), which is a passive haptic system [101]. 

In a study published by Beaudoin et al., knee arthroscopy simulation training with self-learning modules demonstrated an enhancement in skills among untrained participants. Improvements were observed in various areas, procedure time, camera path length, and overall scores [100].

Current active haptic technology, such as Arthro Simbionix (Göteborg, Sweden), which utilizes motors to simulate tactile feedback, falls short in exhibiting adequate face validity or achieving the level of complexity found in passive haptic systems within high-fidelity arthroscopy simulators [102].

A study published by Tronchot et al. showed that skills developed on a hybrid VR simulator could be successfully transferred to bench-top and cadaveric models [103]. This suggests that training with a VR arthroscopy surgical simulator has the potential to safely improve arthroscopic skills in the operating room.

It is currently recognized that VR simulators may play a critical role in the evolution of surgical training, with the ultimate goal of enhancing the quality of patient care [103,104,105]. With surgical and educational applications, an algorithm has been developed that automatically segments the arthroscopic frame to offer surgeons added contextual awareness. The U-NET algorithm is a CNN tailored for biomedical image segmentation. It processes arthroscopic video footage, using it as an input to create a segmented image that delineates the anatomical structures the surgeon observes during the procedure, all in real time [106].

## 7. Ethical Considerations and Critical Aspects

The inception of AI in the mid-20th century aimed to simulate human cognitive abilities; today, ML sophistication enables data gathering, analysis, and problem-solving. In medicine, AI, through algorithms and robotics, influences decision-making and surgical precision. Ethical concerns include patient privacy, algorithm biases, and cybersecurity. AI in medicine, particularly orthopedics, holds transformative potential, aiding in patient care, risk assessment, diagnosis, and surgical procedures. However, rapid advancements bring ethical challenges, necessitating regulatory standards. Ethical concerns include avoiding discrimination, addressing biases in AI algorithms, ensuring patient privacy, and maintaining informed consent. Automation may lead to deskilling and reliance on AI, raising concerns about potential system failures. Cybersecurity and accountability issues also arise, emphasizing the need for continuous monitoring, regulatory updates, and ethical considerations in AI applications in orthopedics. While the idea of fully autonomous medical practice through computer technology seems distant, the evolving field of AI in orthopedics is making significant strides. Currently, AI operates under augmented intelligence, with human oversight crucial for the supervision and monitoring of datasets. Ongoing debates reflect the early stage of development, anticipating more complex ethical dilemmas as AI technology progresses in orthopedics [107].

AI in healthcare, driven by machine learning and deep neural networks, raises significant concerns about patient privacy and bias. The 2015 agreement between DeepMind and the UK National Health Service, later found to violate data protection laws, highlights these privacy issues. Bias in AI, stemming from biased training data, can lead to systematic errors, especially for underrepresented groups. Adversarial attacks pose a serious threat, impacting medical diagnosis, insurance claims, and drug approvals. Regulating AI now may prevent risks but hinder innovation. Solutions include amending regulatory practices, but challenges like deskilling clinicians, data reliance without context, and underestimating medical decision uncertainty remain [7].

## 8. Conclusions and Future Perspectives

**Figure 3:**
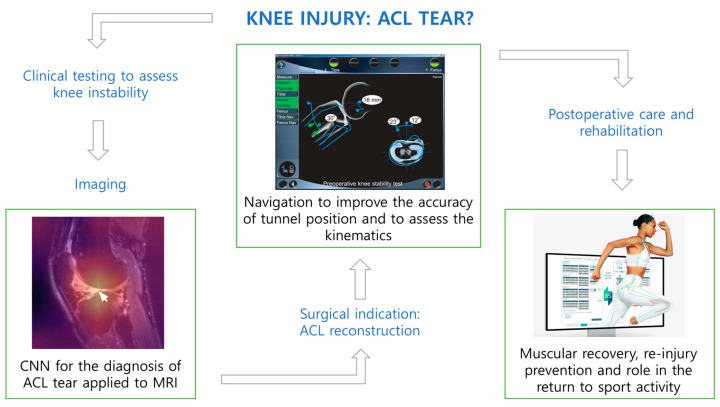
Diagram outlining the main steps in the management of anterior cruciate ligament injury, with the integration of AI tools. (AI = artificial intelligence; CNN = convolutional neural network; ACL = anterior cruciate ligament; MRI = magnetic resonance imaging).

In the management of ACL tears, AI tools can play a role in improving diagnostics, accurately predicting individuals at risk of ACL injury or re-injury, identifying complex anatomic landmarks during surgery, and optimizing pain control and postoperative rehabilitation protocols. Specifically, following an extensive examination of DL in the context of ACL injury diagnosis, it can be asserted that the AI image-assisted diagnostic system, designed for the analysis and processing of multiparametric MRI, proves advantageous for clinical decision-making, alleviates the workload of physicians, improves efficiency, and decreases the likelihood of misdiagnosis. Furthermore, the application of ML and AI in rehabilitation enables a highly personalized and dynamic approach. The continuous learning capability of ML contributes to consistently improving predictions and recommendations, optimizing long-term outcomes. Figure 3 illustrates a diagram with the main steps in the management of ACL injury, from preoperative care to rehabilitation, integrated with AI tools that have broader clinical applications, with the potential for further implementation in the future.

The exponential increase in studies on AI tools applicable to the management of ACL tears promises a significant future impact in its clinical application, with growing attention from orthopedic surgeons.

## Data Availability

Not applicable.
